# Circadian Rhythm Analysis Using Wearable Device Data: Novel Penalized Machine Learning Approach

**DOI:** 10.2196/18403

**Published:** 2021-10-14

**Authors:** Xinyue Li, Michael Kane, Yunting Zhang, Wanqi Sun, Yuanjin Song, Shumei Dong, Qingmin Lin, Qi Zhu, Fan Jiang, Hongyu Zhao

**Affiliations:** 1 School of Data Science City University of Hong Kong Hong Kong China (Hong Kong); 2 Child Health Advocacy Institute Shanghai Children’s Medical Center Shanghai Jiao Tong University School of Medicine Shanghai China; 3 Department of Biostatistics Yale School of Public Health New Haven, CT United States; 4 School of Public Health Shanghai Jiao Tong University Shanghai China; 5 Department of Developmental and Behavioral Pediatrics Shanghai Children’s Medical Center Shanghai Jiao Tong University School of Medicine Shanghai China; 6 Yale Joint Center for Biostatistics Shanghai Jiao Tong University Shanghai China

**Keywords:** wearable device, actigraphy, circadian rhythm, physical activity, early childhood development

## Abstract

**Background:**

Wearable devices have been widely used in clinical studies to study daily activity patterns, but the analysis remains a major obstacle for researchers.

**Objective:**

This study proposes a novel method to characterize sleep-activity rhythms using actigraphy and further use it to describe early childhood daily rhythm formation and examine its association with physical development.

**Methods:**

We developed a machine learning–based Penalized Multiband Learning (PML) algorithm to sequentially infer dominant periodicities based on the Fast Fourier Transform (FFT) algorithm and further characterize daily rhythms. We implemented and applied the algorithm to Actiwatch data collected from a cohort of 262 healthy infants at ages 6, 12, 18, and 24 months, with 159, 101, 111, and 141 participants at each time point, respectively. Autocorrelation analysis and Fisher test in harmonic analysis with Bonferroni correction were applied for comparison with the PML. The association between activity rhythm features and early childhood motor development, assessed using the Peabody Developmental Motor Scales-Second Edition (PDMS-2), was studied through linear regression analysis.

**Results:**

The PML results showed that 1-day periodicity was most dominant at 6 and 12 months, whereas one-day, one-third–day, and half-day periodicities were most dominant at 18 and 24 months. These periodicities were all significant in the Fisher test, with one-fourth–day periodicity also significant at 12 months. Autocorrelation effectively detected 1-day periodicity but not the other periodicities. At 6 months, PDMS-2 was associated with the assessment seasons. At 12 months, PDMS-2 was associated with the assessment seasons and FFT signals at one-third–day periodicity (*P*<.001) and half-day periodicity (*P*=.04), respectively. In particular, the subcategories of stationary, locomotion, and gross motor were associated with the FFT signals at one-third–day periodicity (*P*<.001).

**Conclusions:**

The proposed PML algorithm can effectively conduct circadian rhythm analysis using time-series wearable device data. The application of the method effectively characterized sleep-wake rhythm development and identified the association between daily rhythm formation and motor development during early childhood.

## Introduction

### Background

Wearable devices have been increasingly used in research recently because they can provide continuous objective monitoring of activities and vital signs data such as body temperature and pulse rates [1-3]. In sleep and activity studies, researchers have focused on the actigraphy data generated from wearable devices to study sleep and activity patterns as an alternative to sleep diaries and polysomnography [1,4]. The device usually uses an accelerometer that monitors acceleration in one or more directions, and this wristwatch-like device is often worn on the wrist to record activity continuously for several days. Either the raw data or the transformed activity count data can be used to study sleep-wake patterns and screen sleep disorders [4,5]. Actigraphy not only avoids the subjectivity and bias issues with sleep diaries but also overcomes the drawbacks of polysomnography, such as high costs, in-laboratory setting, intrusive measures, and difficulty in long-term monitoring.

Continuous objective monitoring using wearable devices provides researchers with the opportunity to conduct circadian rhythm studies. Circadian rhythms are endogenous and entrainable biological processes that follow a period of approximately 24 hours, and many physiological phenomena such as sleep-wake patterns, body temperature, and hormone levels exhibit circadian rhythms. For humans, most circadian rhythms are under the control of the pacemaker located in the suprachiasmatic nuclei in the anterior hypothalamus of the central nervous system, and suprachiasmatic nuclei accept environmental information such as the light and dark cycle to adjust the 24-hour cycle [6]. However, 24-hour human circadian rhythms are not mature at birth, when the predominant rhythm is ultradian, and the circadian rhythms of sleep-wake cycles and body temperature gradually develop during the first year after birth [6-9]. Many studies have investigated how circadian rhythms develop through childhood into adolescence and adulthood and how they are related to health issues such as sleep problems, mental problems, and disease risks, to name a few [8,10-14]. It is noteworthy that the development of circadian rhythms during early childhood is associated with disease risk factors and can affect both childhood and adult life [8]. Therefore, it is important to conduct circadian rhythm studies to gain a thorough understanding of the formation and consolidation of daily activity rhythms during early development as well as the association between the changes in daily rhythms and health conditions.

Actigraphy data generated from wearable devices have been validated to provide reliable information on sleep and circadian rhythms [15]. However, the analysis of time-series data from actigraphy remains a major obstacle for researchers. Current major statistical methods are either parametric, based on cosinor analysis, or nonparametric [16-21]. These methods do not specifically focus on periodic information and are not specifically suitable for populations whose sleep-wake rhythms are not sinusoidal, such as patients with Circadian Rhythm Sleep Disorder, or not mature, such as young infants and toddlers [6-10,12]. Therefore, traditional approaches targeting normal daily rhythms might not work because detailed activity rhythms cannot be captured. There is a need to develop an appropriate methodology to extract periodic information and study detailed circadian patterns of all populations to better characterize daily rhythms.

### Objective

In this paper, we propose a Penalized Multiband Learning (PML) approach that can complement current methods to characterize daily rhythms based on periodic information in time-series wearable device data. PML extracts periodic information using the Fast Fourier Transform (FFT) algorithm and then performs penalized selection based on regularization, a classic approach used in machine learning, to identify dominant periodicities and further characterize daily rhythms [22,23]. In this paper, we first present the proposed PML approach in detail and discuss its usefulness and advantages compared with other methods. Subsequently, we present an application of the method to early childhood wearable device activity data, in which we characterize the formation and consolidation of sleep-activity rhythms and further study its association with physical development during early childhood.

## Methods

### Data

**Figure 1:**
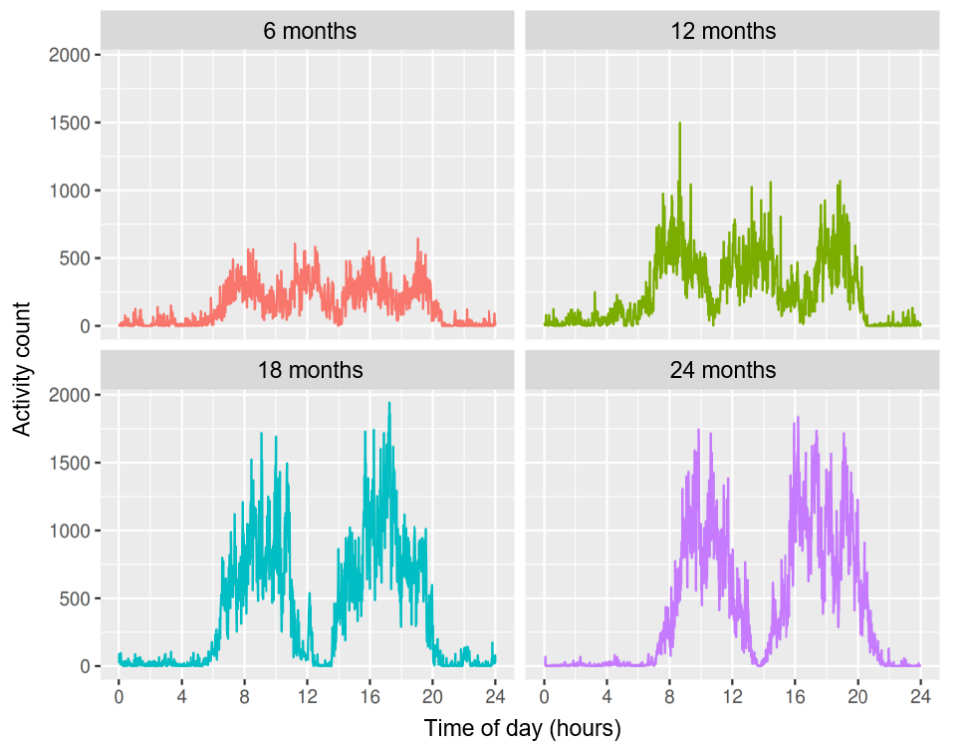
The activity plots for participant ID 17 at 6, 12, 18, and 24 months, with activity counts averaged across 7 days at each time point.

The study participants were 262 healthy newborns recruited in 2012-2013 by the Shanghai Children’s Medical Center, Shanghai, China. Actiwatch data were collected at ages 6, 12, 18, and 24 months, with 159, 101, 111, and 141 participants at each time point, respectively; not all participants from the cohort participated each time. The infants and toddlers were required to wear Actiwatch 2 (Philips Respironics Mini-Mitter) on the ankle for 7 consecutive days. Wearing such devices on the ankle is commonly recommended for young infants or toddlers [24]. The Actiwatch 2 uses a piezoelectric sensor to detect accelerations between 0.5 and 2.0 g with a frequency response range between 0.35 and 7.5 Hz, and activity counts summarize the accelerations over each epoch. The data output format for Actiwatch 2 was configured to be the activity count per 1-minute epoch. On the basis of sleep diaries and activity plots for each individual, the days showing nonwear periods with straight lines of zero activity counts were removed. Nonwear periods can be differentiated from sedentary behaviors or sleep because the former gives almost all zero activity counts, whereas the latter gives nonzero activity counts every now and then. Figure 1 shows the activity plots for participant ID 17, and it can be seen that at 6 months, low and high activities were intermittent during the day, suggesting multiple daytime naps, whereas near-zero activity levels at night suggest long nighttime sleep. At 12 months, 3 activity peaks, one morning nap, and one afternoon nap can be identified. At 18 and 24 months, 2 activity peaks formed and stabilized, showing only one afternoon nap. The daily activity rhythm developed and stabilized as the infant grew. In addition to Actiwatch data, demographic information and family information were collected at baseline, such as child’s sex, child’s date of birth, parents’ age, child’s birth weight and body length, parents’ height and weight, parents’ educational levels and working status, and family income. The Peabody Developmental Motor Scales-Second Edition (PDMS-2) was used to assess early childhood physical development at 6, 12, 18, and 24 months [25]. The institutional review board of the Shanghai Children’s Medical Center, Shanghai Jiao Tong University, approved the study (approval number: SCMCIRB-2012033). The parents of the children who participated in the study provided written informed consent.

### FFT Algorithm

**Figure 2:**
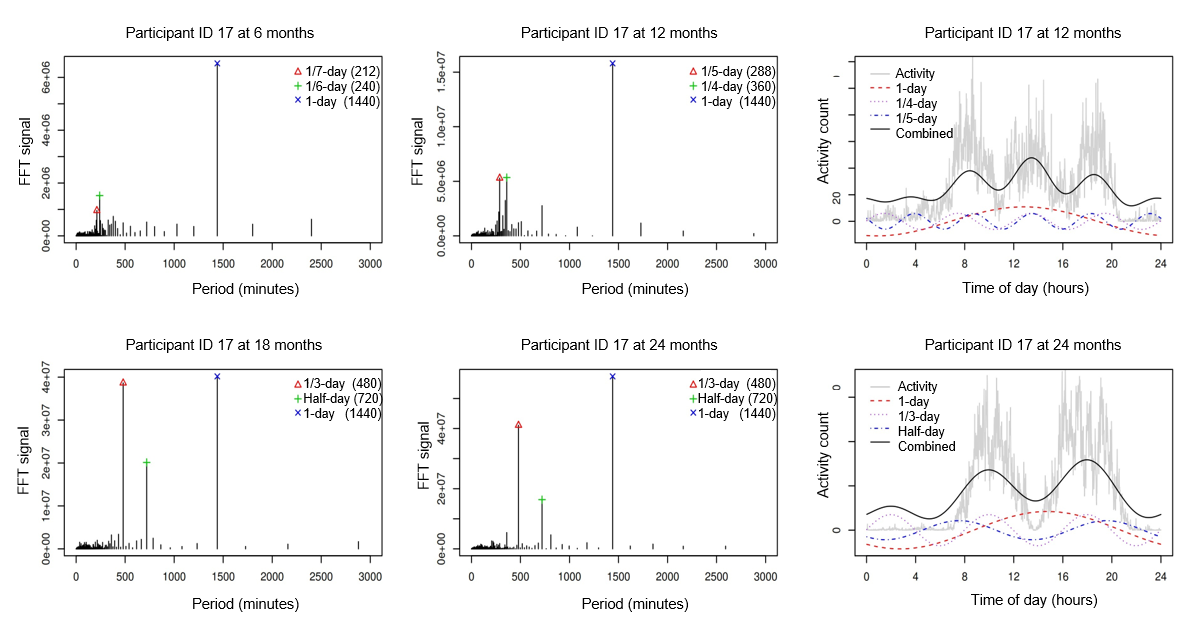
Four panels on the left: Fast Fourier Transform results for participant ID 17 at 6, 12, 18, and 24 months. Two panels on the right: top three periodicities and the combined periodicities plotted on 1-day observation for participant ID 17 at 12 and 24 months. FFT: Fast Fourier Transform.

To describe the consolidation of sleep-activity rhythms during early childhood, we used periodic information to characterize the daily rhythms. Specifically, we used the FFT algorithm to convert time domain signals into a frequency domain spectrum to extract periodic information. We analyzed the original data to allow for non–24-hour sleep-wake rhythm detection. Figure 2 shows the FFT results for participant ID 17 at each age. The 1-day periodicity was the most dominant at all time points. The one-fifth–day and one-fourth–day periodicities can be identified at 12 months. The half-day and one-third–day periodicities did not become dominant until 18 and 24 months. It is noteworthy that each periodicity is not interpreted alone; rather, the periodicities are combined to understand the overall pattern. As suggested in the 2 right panels of Figure 2, the combined one-fifth–day and one-fourth–day periodicities form a three-peak, two-nap pattern at 12 months. Similarly, the combined half-day and one-third–day periodicities exhibited a two-peak, one-nap pattern at 18 and 24 months. Therefore, the combination of dominant periodicities can be used to capture the main sleep-activity patterns at each age and describe the gradual consolidation of daily rhythms in early childhood development.

### Identification of Dominant Periodicities

The PML algorithm is as follows: let matrix *X* ∈ R*^n^*^×^*^p^* denote the FFT results, where denotes the number of individual observations and denotes the number of periodicities from FFT. Specifically, *X* = (*x*_1_,*x*_2_,...,*x_p_*), where *x_j_* is the vector of length *n* for the *j*th periodicity.

Let Θ be the diagonal matrix selecting columns from *X* such that 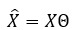 and 0≤*θ_j,j_*≤1, *j*=1*,...,p:*



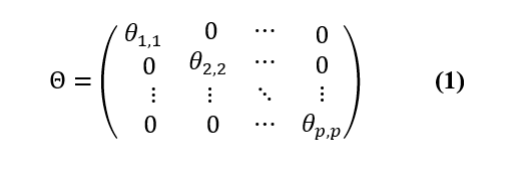



Θ identifies columns of dominant periodicities from *X* in such a way that dominant periodicities corresponding to nonzero *θ_j,j_*’s are selected. We minimized the squared Frobenius norm 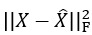, which is the sum of the squared elements of the matrix. Using the properties of the Frobenius norm, we obtained the following:



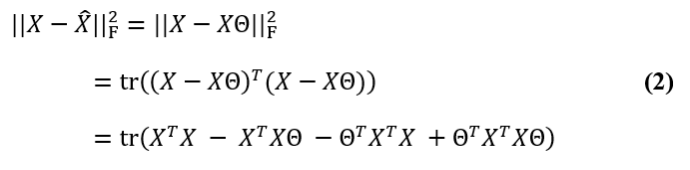



As *X^T^X* is fixed, it is equivalent to minimize as follows:



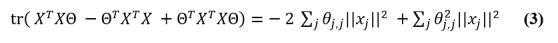



To estimate Θ and identify dominant periodicities, we used a penalized selection method similar to Lasso, a widely used method in shrinkage and selection of a subset of features in regression models and machine learning approaches [23]. In regression analysis, the Lasso penalty is most effective in selecting a few important features while suppressing the regression coefficients of other nonselected features to 0 [23]. In our case, the Lasso penalty served to select a few dominant periodicities through diagonal elements of Θ instead of regression coefficients. Furthermore, we added an elastic net–like penalty term to the Frobenius norm, namely a combination of L1 and L2 norms [22]:



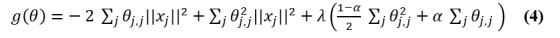



where λ is the turning parameter and α controls the balance between the L1 and L2 norms. Note that *θ_j,j_*’s is nonnegative; thus, we do not need to take the absolute value for the L1 norm. By setting λ large enough, all diagonal elements of Θ, namely all *θ_j,j_*’s are suppressed to zero, and no periodicities are selected. As λ decreases, some *θ_j,j_*’s become nonzero, and they correspond to the most dominant periodicities that are selected sequentially according to their dominance.

To minimize *g*(*θ*), we took the partial derivative of *g*(*θ*) with respect to each 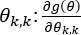 = –2||*x_k_*||^2^ + 2*θ_k,k_*||*x_k_*||^2^ + (1-*a*)*λθ_k,k_* + *aλ*,which is convex and subject to the constraint 0≤*θ_k,k_*≤1. Thus, we have the following:



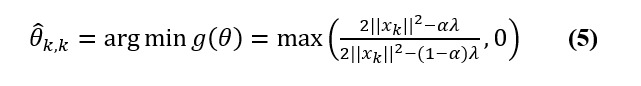



If we only have the L1 penalty, then α=1 and 
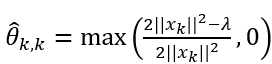
. In our case, we used the Lasso L1 penalty alone and trained λ because we wanted to select the most important periodicities while suppressing other periodicities to 0. However, we still kept the L2 norm in the original model as an option because it might be helpful in future tasks such as prediction, classification, and reconstruction of curves.

We used the mean squared error (MSE), which is equivalent to the squared Frobenius norm 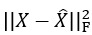, as the criterion for choosing λ and the number of nonzero *θ_j,j_*’s (the number of dominant periodicities selected), as well as to evaluate the variability that was not explained by the selected periodicities. We did not choose cross-validation because the results showed that the test data set error curve was monotonous. We trained λ from 
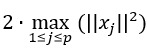
 to 0 because λ = 
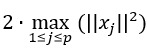
 suppresses all *θ_j,j_*’s to and λ=0 gives no penalty. By decreasing λ, we identified dominant periodicities sequentially and characterized the daily sleep-activity rhythm at each age. An R package named PML was developed [26] for the implementation of the PML algorithm [27].

### Comparison With Other Methods

To rigorously conduct statistical tests and select significant periodicities, we applied the Fisher test in harmonic analysis [28]. It is a sequential test for ordered statistics, and periodicities are first ordered and then tested for significance. If one periodicity is statistically significant, the next one will be tested further. Otherwise, the sequential test will be stopped. At each step, the critical value at which to declare statistical significance was different. In some studies, the method may not be implemented correctly; therefore, we included the sequential test in the R package for easy implementation. As we performed multiple testing, Bonferroni correction was used to adjust the *P* values. If we conduct the tests at significance level α, we reject the null hypothesis if *P* value≤α*/p*, where *p* is the number of periodicities, and conclude that the periodicity is significant.

To evaluate the effectiveness of the PML algorithm, we compared it with the autocorrelation of the standard approach. The autocorrelation *r_k_* is calculated between activity measurements with a time lag *k,* and the coefficient *r_24_* denoting a 24-hour time lag is of primary interest in circadian studies [29]. *r_k_* ranges between –1 and 1, and a *k*-hour periodic pattern can show a higher value of the correlation coefficient *r_k_*. In the plot of *r_k_* against the time lag *k*, a peak around *k*=24 can be observed when there is a dominant circadian pattern of 24-hour periodicity. We plotted autocorrelation against the time lag to compare the autocorrelation method with our algorithm.

### Association Between Daily Rhythms and Motor Development

Next, we conducted linear regression analysis to study the association between the consolidation of daily activity rhythms and early childhood physical development. PDMS-2 is considered an early childhood developmental assessment tool, and the score is used as the outcome. If the PDMS-2 total motor standard scores are found to be associated with daily rhythm features, the standard scores for the subtests, including stationary, locomotor, object manipulation, grasping, and visual-motor integration as well as gross motor and fine motor, are used as the outcome to examine which specific subcategory is associated. Gross motor represents the overall performance on stationary, locomotion, and reflexes (6 months) or object manipulation (12, 18, and 24 months) for infants, and fine motor represents the overall performance on grasping and visual-motor integration.

The FFT signals at dominant periodicities identified by the PML were used as daily rhythm features and considered covariates in the model. In addition to periodic features, demographic information and family information as potential confounders were also considered in the model. Backward selection was used in the model-fitting process. Although some variable (denoted as variable A here) may seem to be statistically significant in the complete model, after removal of insignificant variables in the variable selection process, variable A may become insignificant. In such cases, variable A is removed in the final model to achieve parsimony.

Linear regression analysis was conducted at 6, 12, 18, and 24 months to study the association between daily rhythms and motor development. For the final model comparison, *r*^2^, which measures the proportion of variance in the outcome explained by the model, and the adjusted *r*^2^, which modifies *r*^2^ based on the number of predictor variables, were also calculated. All statistical analyses were conducted using R version 3.3.2 (R Foundation for Statistical Computing).

## Results

### Identification of Dominant Periodicities

**Figure 3:**
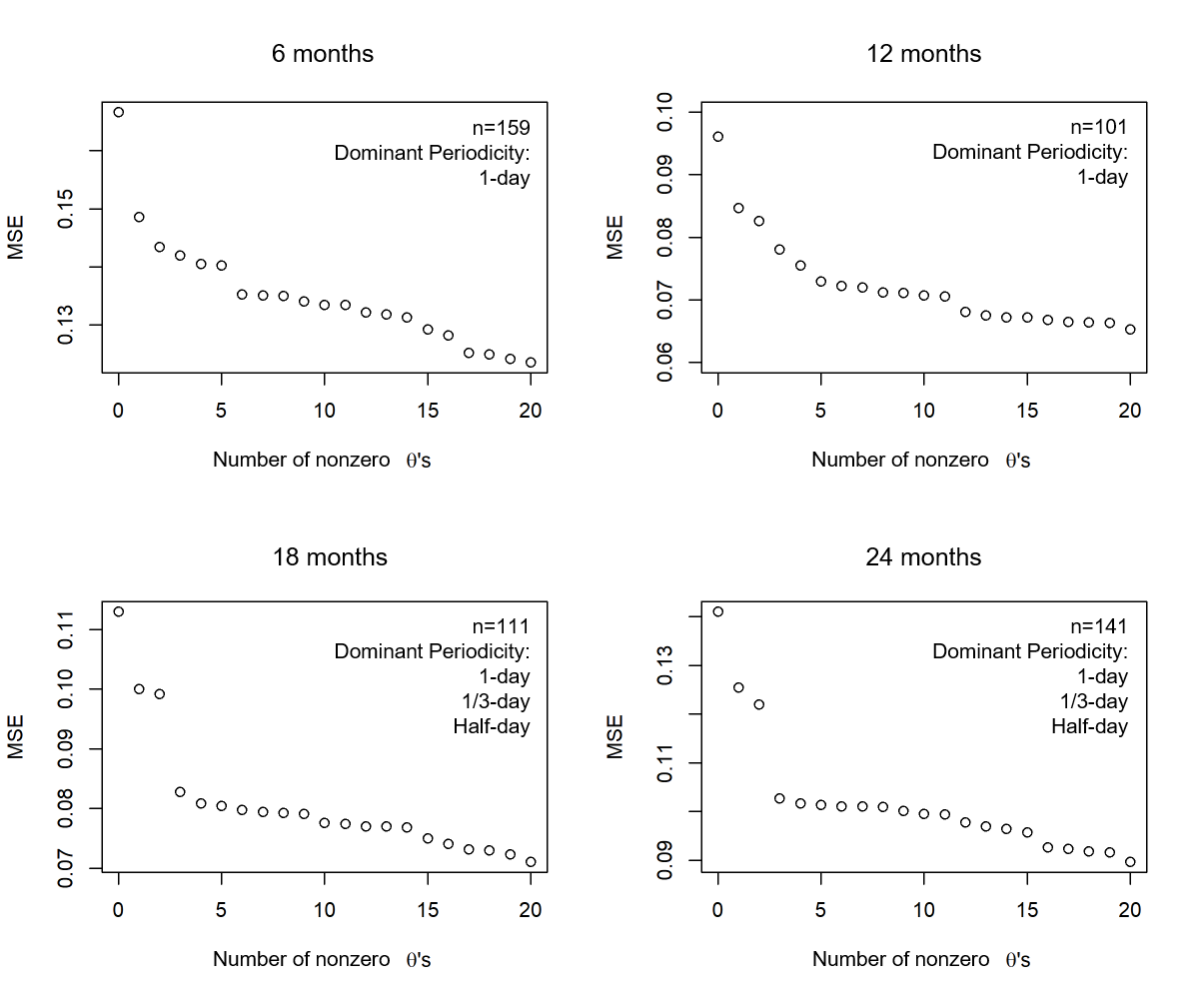
Mean squared error plotted against the number of nonzero θ’s as the penalty term λ decreased at 6, 12, 18, and 24 months. MSE: mean squared error.

As shown in Figure 3, at each age, we plotted the MSE against the number of nonzero *θ*’s. Specifically, we plotted only the points where the number of nonzero *θ*’s (periodicities selected) increased as the penalty term λ decreased. For 6 months and 12 months, the first harmonic at 1-day periodicity is the most dominant because we can observe a large dip in the MSE when the first periodicity is selected, whereas the periodicities that are further selected do not cause the same level of decrease. For 18 and 24 months, the first three periodicities of 1 day, one-third day, and half day are the most dominant, and selecting the first three can lead to a relatively large decrease in the MSE. The results indicate that during the first year, only one-day periodicity was formed and stabilized in the infant population. The sleep-activity rhythm did not stabilize until 18 months, showing the pattern of 1 nighttime sleep, 1 daytime nap, and 2 daytime activity peaks.

The Fisher tests yielded similar results. As shown in Table 1, only 1-day periodicity was significant at 6 months (*P*<10^–5^) because for infants at this stage, sleep-activity patterns have already adjusted to a 24-hour cycle. However, daytime activities have not yet been stabilized and variations exist across days. At 12 months, the 4 periodicities were significant (*P*<10^–5^). This is because infants’ sleep-activity patterns start to stabilize, but there are variations across individuals: some take one nap in the afternoon, whereas others take two naps, one in the morning and one in the afternoon. The one-nap pattern can be captured by the one-third–day periodicity, whereas the two-nap pattern can be captured by the one-fourth–day periodicity, as shown in Figure 2. Furthermore, at 18 and 24 months, three periodicities were significant (*P*<10^–5^): 1 day, half day, and one-third day, indicating the final consolidation of daily sleep-activity rhythms with only one daytime nap in the afternoon. In addition, the proportions of variance explained by the half day and one-third day periodicities at 18 months and 24 months were approximately the same, both higher than those at 12 months.

**Table 1:** Significant periodicities at 6, 12, 18, and 24 months, with the corresponding proportions of variances among all Fast Fourier Transform signals and *P* values.

Age (months)	Proportions of variance	Period		*P* value
		Minutes	Days	
6	0.0110	1440	One	6.59×10^–8^
12	0.0120	1440	One	1.75×10^–8^
12	0.0068	720	Half	3.75×10^–7^
12	0.0056	360	One-fourth	2.82×10^–7^
12	0.0045	480	One-third	6.49×10^–6^
18	0.0130	1440	One	1.46×10^–9^
18	0.0085	480	One-third	1.87×10^–10^
18	0.0083	720	Half	5.02×10^–15^
24	0.0130	1440	One	1.97×10^–9^
24	0.0086	480	One-third	1.17×10^–10^
24	0.0080	720	Half	3.78×10^–14^

### Comparison With Autocorrelation

**Figure 4:**
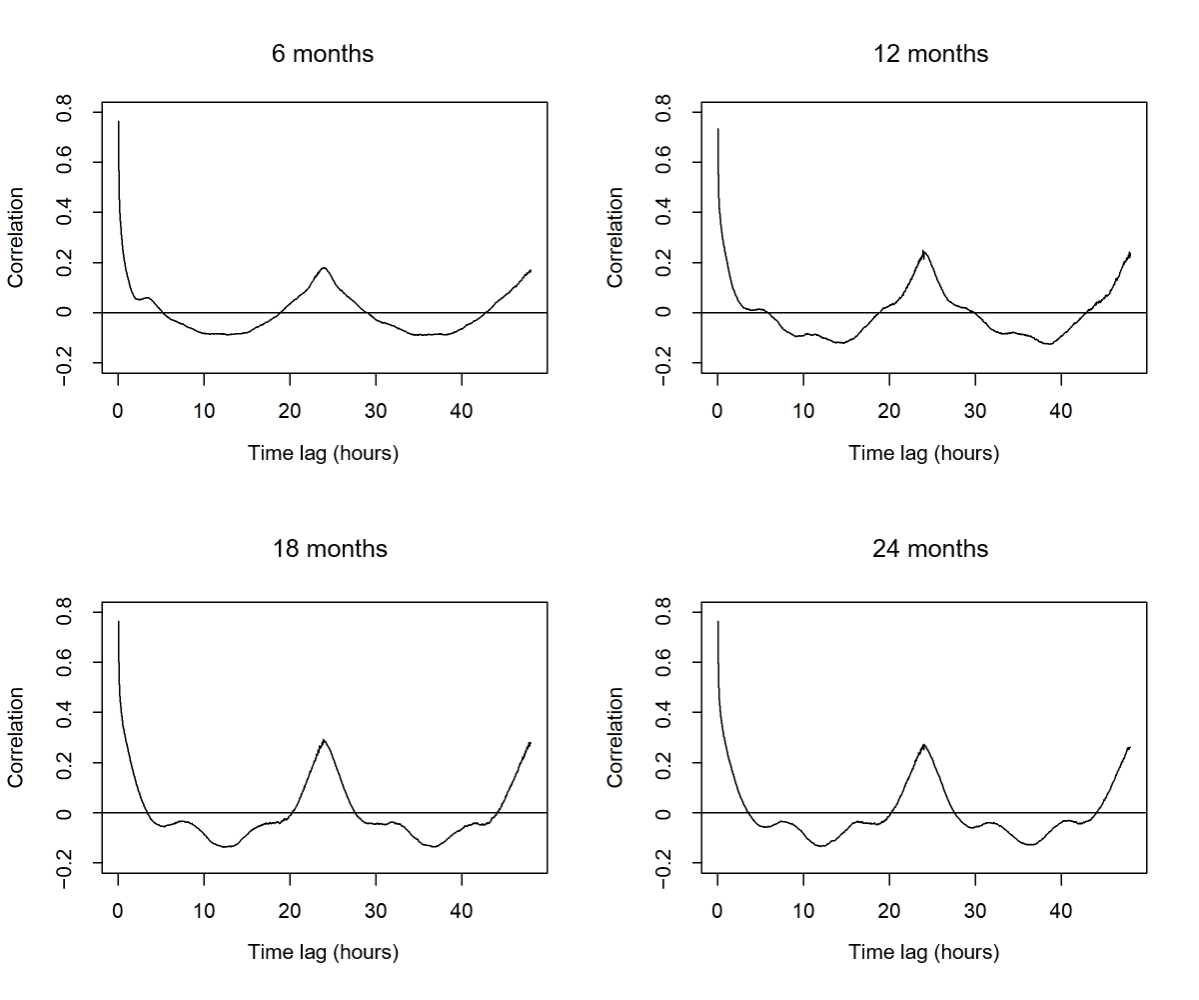
Estimated autocorrelation against time lags at 6, 12, 18, and 24 months.

To compare the PML algorithm with autocorrelation, the plot of correlation estimates against time lags is shown in Figure 4. The circadian rhythm at 24 hours can be observed at all time points because the peaks of estimated correlation are at time lags between 23.8 hours and 24.3 hours. We can also observe some local maximal correlation estimates at other time lags: 3.3 hours at 6 months, 4.7 and 10.7 hours at 12 months, 7.5 hours at 18 months, and 7.5 and 16.3 hours at 24 months. Although 3.3 hours at 6 months may seem reasonable because of the infant feeding schedule, other cycles are difficult to explain [30,31]. Autocorrelation estimates can be biased because of the presence of multiple periodicities, and researchers generally use these estimates to verify the most dominant periodicity such as 24 hours. Thus, from the autocorrelation plots, the most dominant 24-hour rhythm that yields the global maximal correlation estimate can be identified at each age, and this dominant periodicity was also identified by PML.

### Association Between Daily Rhythms and Motor Development

The summary of the PDMS-2 standard scores for each category is presented in Table 2.

As shown in Table 3, at 6 months, the PDMS-2 total motor scores were found to be associated with the assessment seasons (*P*<.001). Infants receiving the PDMS-2 assessment in winter and spring tended to have lower PDMS-2 total motor scores than those assessed in summer and autumn. At 12 months, the PDMS-2 total motor scores were associated with both seasons and FFT signals: infants assessed in summer tended to have higher PDMS-2 total motor scores, and infants with higher FFT signals detected at one-third–day and half-day periodicities also tended to have higher PDMS-2 total motor scores (*P*<.001 and *P*=.04, respectively). *r*^2^ was 0.25, and the adjusted *r*^2^ was 0.21. At 18 and 24 months, no association was identified between the PDMS-2 total motor scores and any other variables.

**Table 2:** The PDMS-2^a^ standard scores for the subtests, gross motor, fine motor, and total motor.

PDMS-2 category	Standard scores, mean (SD)			
	6 months (n=246)	12 months (n=225)	18 months (n=192)	24 months (n=170)
Reflexes	10.56 (1.02)	—^b^	—	—
Stationary	9.67 (1.44)	9.48 (1.00)	9.94 (0.36)	9.03 (1.38)
Locomotor	10.09 (1.08)	8.82 (1.76)	9.26 (1.24)	8.61 (1.70)
Object manipulation	—	9.92 (0.78)	9.49 (1.16)	8.74 (1.33)
Grasping	10.69 (1.02)	11.08 (1.44)	9.55 (0.78)	9.99 (0.98)
Visual-motor integration	11.19 (1.20)	10.69 (1.13)	11.04 (1.74)	9.79 (1.74)
Gross motor	30.34 (2.72)	28.25 (2.65)	28.63 (2.21)	26.16 (3.17)
Fine motor	21.86 (2.02)	21.83 (2.17)	20.67 (1.93)	19.80 (2.22)
Total	52.24 (4.18)	50.23 (5.05)	49.43 (3.19)	45.98 (4.69)

^a^PDMS-2: Peabody Developmental Motor Scales-Second Edition.

^b^Reflexes is only for 6-month infants, and object manipulation is only for 12-, 18-, and 24-month infants.

**Table 3:** Linear regression analysis of Peabody Developmental Motor Scales-Second Edition total motor standard scores by season and Fast Fourier Transform signals at 6 months and 12 months, respectively.

Time and variable		Estimate (SE)	*t* test (*df*)	*P* value
**6 months**				
	(intercept)	50.10 (0.56)	89.52 (155)	<.001
	spring	0.48 (0.85)	0.56 (155)	.58
	summer	4.48 (0.82)	5.43 (155)	<.001
	autumn	4.06 (0.89)	4.54 (155)	<.001
**12 months**				
	(intercept)	45.52 (1.29)	35.25 (96)	<.001
	summer	1.66 (1.58)	1.05 (96)	.30
	half day^a^	0.19 (0.09)	2.10 (96)	.04
	one-third day^a^	0.31 (0.09)	3.55 (96)	<.001
	summer: one-third day^a^	-0.48 (0.16)	−3.00 (96)	.004

^a^The Fast Fourier Transform signals were multiplied by 10,000 in regression models, so that the estimated effect sizes were for every 10,000-unit increase in the Fast Fourier Transform signals.

As PDMS-2 total motor scores are associated with FFT signals at 12 months, further linear regression between each subtest score and FFT signal was also examined. As shown in Table 4, subtests for stationary and locomotion as well as gross motor and fine motor were found to be associated with the one-third–day periodicity. The gross motor represents the overall performance on the three subtests of stationary, locomotion, and object manipulation for infants at 12 months, and because the association of FFT signals at one-third–day periodicity with stationary and locomotion subtests is strong, it is expected that the association of FFT signals at one-third–day periodicity with gross motor is also strong. *r*^2^ and the adjusted *r*^2^ were 0.05 and 0.04 for the stationary model, 0.23 and 0.20 for the locomotion model, 0.21 and 0.17 for the gross motor model, and 0.15 and 0.11 for the fine motor model, respectively.

**Table 4:** Linear regression analysis of Peabody Developmental Motor Scales-Second Edition standard scores by season and Fast Fourier Transform signals at 12 months: stationary and locomotion subtests and gross motor and fine motor as the outcome, respectively.

Category and variable^a^			Estimate (SE)		*t* test (*df*)		*P* value
**Stationary**							
	(intercept)	9.23 (0.19)		47.9 (99)		<.001	
	one-third day	0.04 (0.02)		2.17 (99)		.03^b^	
**Locomotion**							
	(intercept)	7.16 (0.43)		16.48 (97)		<.001	
	summer	0.71 (0.75)		0.94 (97)		.35	
	one-third day	0.19 (0.04)		4.61 (97)		<.001^b^	
	summer: one-third day	–0.13 (0.07)		–1.93 (97)		.06^c^	
**Gross motor**							
	(intercept)	26.41 (0.63)		42.03 (97)		<.001	
	summer	0.57 (1.09)		0.52 (97)		.60	
	one-third day	0.24 (0.06)		3.97 (97)		<.001^b^	
	summer: one-third day	–0.17 (0.10)		–1.73 (97)		.09^c^	
**Fine motor**							
	(intercept)	21.08 (0.5)		42.15 (97)		<.001	
	summer	0.34 (0.86)		0.39 (97)		.70	
	one-third day	0.11 (0.05)		2.36 (97)		.02^b^	
	summer: one-third day	–0.15 (0.08)		–1.89 (97)		.06^c^	

^a^The Fast Fourier Transform signals were multiplied by 10,000 in regression models so that the estimated effect sizes were for every 10,000-unit increase in the Fast Fourier Transform signals.

^b^Statistical significance level at α=.05.

^c^Statistical significance level at α=.10.

## Discussion

### Method Evaluation

The PML approach is very effective in studying daily activity rhythms among infants and toddlers. At 6 and 12 months, the dominant 1-day periodicity suggests the formation of the 24-hour cycle. At 18 and 24 months, the combination of the dominant 1-day, one-third–day, and half-day periodicities forms a consolidated daily activity pattern with 2 activity peaks during the day and 1 afternoon nap. PML not only effectively identified population-level dominant periodicities, but also characterized sleep-activity patterns without complex functional analysis. PML can complement current methods for circadian rhythm analysis and is especially useful for populations whose daily rhythm patterns are nonsinusoidal and irregular. On the other hand, because PML is applicable to time-series data with a nature similar to that of actigraphy, the application of PML can be extended to other types of circadian rhythm studies using information such as body temperature and hormone data to study and characterize daily rhythms effectively.

### Comparison With Other Methods

In comparison, the Fisher test in harmonic analysis tends to identify many significant periodicities unless a stringent threshold is used for statistical significance. In this study, we used the Bonferroni correction to adjust for multiple testing and used a significance level of 10^–5^ to select the periodicities, even though we did not conduct as many statistical tests simultaneously. In sequential testing procedures, as was the case in our study, people often use less-conservative multiple testing correction methods such as the Benjamini-Hochberg procedure [32]. We chose the most stringent threshold to avoid selecting too many periodicities that are not helpful in characterizing daily activity patterns at each age.

We also compared our PML algorithm with the standard approach autocorrelation. Plots of correlation estimates against time lags are useful for identifying the correlation peak at 24 hours but not for shorter periods of rhythmicity. This is because the estimation of correlation can be biased because of the presence of multiple periodic rhythms, and the identification of multiple periodicities by simple calculation of autocorrelation may not be accurate. Therefore, the standard approach using autocorrelation is effective in confirming the most dominant 24-hour periodicity but is not as effective in identifying other periodicities, which the PML algorithm can achieve.

Other standard approaches such as periodograms and cosinor analysis were not used in this study because there are, in fact, connections between PML and the two methods. It is important to point out their connections as well as differences. Among periodograms, the Fourier, Enright, and Lomb-Scargle periodograms are commonly used [18]. Both our PML algorithm and the Fourier periodogram use Fourier analysis to identify dominant periodicities, but the PML algorithm uses a shrinkage method in machine learning to identify dominant patterns in the population, whereas Fourier periodograms are focused on individuals to manually identify dominant periodicities based on individual plots. The Enright periodogram, although suitable for equidistant activity measurements in our scenario, may not be applicable here because it requires ≥10 days of data [18]. In addition, the estimation method only holds when there is one periodic component, but in our case, the presence of multiple periodic components may have attenuated the results [18]. The Lomb-Scargle periodogram is a modification of Fourier analysis to accommodate unevenly spaced data or missing data. As our data do not have this issue, the Lomb-Scargle periodogram is equivalent to the Fourier periodogram in our case. Compared with the PML algorithm, the Fourier periodogram involves more manual work to generate periodograms for each individual and visually identify dominant periodicities, whereas PML is more automated and more effective in studying the population as a whole and further identifying the periodicities that are characteristic of the population. In addition, researchers often use periodograms to validate the most dominant periodicity such as 24 hours but do not specifically examine information on secondary dominant periodicities or use periodic curves to reconstruct or approximate activity patterns, even though the connection between dominant frequencies or periodicities and functional curves can be made and the periodic information can be fully used. Therefore, the PML algorithm makes full use of the information from more than one dominant periodicity and links the FFT results in the frequency domain with their corresponding cosine curves to effectively characterize activity patterns.

For cosinor analysis, one may recall that the FFT results consist of real parts and imaginary parts that correspond to cosine curves and sine curves, respectively; thus, FFT is equivalent to fitting the cosine model. We fitted cosine models to the activity data with 1 to 3 cosine curves at dominant periodicities identified by the PML algorithm. Even though the estimated amplitudes for the cosine curves are different from the amplitudes in the FFT results, the Pearson correlation between the cosine coefficients and the FFT signals of the same periodicity is 1, indicating equivalence. Although the final results are equivalent, the procedures are different. For cosinor analysis, if prior knowledge is available, simple least squares methods can be used to fit the model [33]. However, if there is no prior knowledge of periodic information, the least squares method cannot be used because the dominant periodicity needs to be estimated first, and the cosinor model can no longer be linearized in its parameters. One has to either start from an initial guess and use iterative procedures to minimize the residual sum of squares or use simulated annealing alternatively to fit the model, the process of which can be exhaustive [34-36]. In comparison, without prior knowledge of the dominant periods, the PML algorithm based on shrinkage in machine learning is still easy to implement without computational burden in extracting periodic information, and the results are as effective as the cosinor model to characterize daily activity patterns using cosine curves.

In summary, the proposed PML algorithm is effective in extracting periodic information, identifying dominant periodicities, and characterizing activity patterns. In the presence of multiple periodicities, PML does not have the estimation problem that autocorrelation encounters. To identify dominant periodicities, PML uses shrinkage in machine learning methods that can help researchers avoid manual work in periodograms, which require individual plots and visual identification. PML can also characterize activity patterns by making full use of the cosine curves represented by FFT signals and avoiding the computationally intensive process of choosing and fitting cosinor models when prior knowledge of the dominant periodicities is not available.

### Sleep-Activity Rhythm Characterization

Our study confirms previous findings that infants already form 24-hour sleep-wake cycles at 6 months due to entrainment by cyclical changes in the environment, whether it is due to light-dark cycles or maternal rest-activity cycles [6,9,10,37,38]. It is noteworthy that although 24-hour cycles are formed, sleep-activity patterns over the 24-hour period are not stabilized: infants often take multiple naps at different times of the day, and their daily activity patterns vary across days and across individuals.

Our study indicates that from 6 months to 12 months, the infant sleep-activity pattern gradually develops: some infants take only 1 afternoon nap, whereas others take two naps: one in the morning and one in the afternoon. Strong FFT signals at one-third–day periodicity capture two-peak, one-nap activity patterns, whereas strong FFT signals at one-fourth–day periodicity capture three-peak, two-nap patterns. The results are in line with those of previous sleep studies which indicated that the duration of nighttime sleep gradually lengthens and sleep patterns become increasingly consolidated during the first year after birth [12,39].

Although the timing for the stabilization of infant sleep-activity patterns varies across individuals, by the time infants reach the age of 18 months, their daily activity patterns have consolidated into a predominant nighttime pattern with 1 afternoon nap only, which can be obtained by combining the 3 dominant periodicities at 1 day, half day, and one-third day. The consolidation of sleep-activity patterns is confirmed by increased FFT signals at half-day and one-third–day periodicities and decreased FFT signals at other periodicities compared with previous ages. The results for 24 months remained the same as those at 18 months, showing no changes and confirming further that sleep-activity patterns are consolidated by 18 months and are stable from that age onward.

In our study, the 3-hour periodicity, normally for feeding behaviors, was not detected, and there might be 2 reasons for this. First, in the feeding guidelines for infants, the feeding of infants aged 6-8 months is advised to be 5-6 times in 24 hours, less frequent than the 3-hour (8 times) schedule, and it is advised that infants aged 12-24 months should have 3 meals with family and have additional snacks 2-3 times [30,31]. Infants aged below 6 months may have a more frequent feeding schedule, but our activity data were collected at 6 months or later. Second, there might be desynchronization between the feeding schedules and activity patterns. Although infants aged 6 months might be fed 5-6 times per day, they do not nap or sleep 5-6 times within the same time frame. We referred to the sleep diaries recorded by the parents as a reference for napping information. Most infants aged 6 months have 1 to 2 naps in the morning and 1 nap in the afternoon. Infants aged 12 months generally have 1 or no naps in the morning and 1 nap in the afternoon. Most infants aged 18 months and 24 months have 1 nap in the afternoon. Therefore, sleep-activity patterns are desynchronized with feeding schedules because feeding behaviors might not dominate infant sleep-activity patterns at this age. For the aforementioned reasons, feeding cycles such as 3-hour periodicity were not detected in our data.

### Association Between Daily Rhythms and Early Childhood Development

Using FFT signals at dominant periodicities identified by PML, we were able to find an association between the consolidation of sleep-activity rhythms and early childhood motor development. At 6 months, the association between PDMS-2 total motor scores and assessment seasons may be explained by the different number of layers of clothing worn by infants in different seasons. In winter, infants are likely to wear many layers of clothing, which may restrict their behaviors and result in suboptimal performance compared with infants wearing light clothes and taking the PDMS-2 assessment in summer. As a result, infants aged 6 months who were assessed in summer and autumn obtained relatively higher PDMS-2 scores than infants assessed in winter and spring.

At 12 months, after controlling for the assessment seasons, stronger FFT signals at half-day and one-third–day periodicities were generally associated with higher PDMS-2 scores. The period of 12 months is critical for sleep-activity rhythm consolidation, which was captured by the growing FFT signals at half-day and one-third–day periodicities. It is noteworthy that all the infants at this age had strong FFT signals at 1-day periodicity, indicating that they exhibited 1-day periodicity in their sleep-activity patterns and that their 24-hour periodic activity patterns tended to be stabilized. As a result, there was not much variation in the strength of 1-day periodicity, which might not explain much of the variability in the PDMS-2 scores among individuals. In comparison, the activity pattern over the 24-hour period was not consolidated, and the activity pattern could be characterized by the one-third–day and half-day periodicities. The larger variability in the strength of one-third–day and half-day periodicities can describe the degree to which the daily sleep-activity pattern is consolidated, which is further associated with child development as evaluated by the PDMS-2 scores. The infants with a more consolidated activity pattern tended to have better motor development evaluations. In addition, activity rhythm consolidation is strongly associated with the subcategories of locomotion and stationary, which belong to the gross motor and measure how the large muscle system is used to move from place to place or assume a stable posture when not moving. Therefore, we obtained new insights into early childhood development that the degree to which the sleep-activity pattern is consolidated at 12 months is associated with infant motor development and with large muscle system development in particular.

At 18 and 24 months, the PDMS-2 scores were not associated with the season, FFT signals, or any other variables in our data set. Most of the toddlers had stabilized daily activity patterns with strong periodic rhythmicity at this age. FFT signals as characteristics of sleep-activity rhythms were no longer associated with the PDMS-2 scores, and this is likely because the critical age at which the daily activity rhythm stabilizes had passed.

### Limitations and Future Work

One limitation of our study is that we collected Actiwatch data every 6 months, and thus we were not able to capture more detailed monthly changes over the period. Future work may collect Actiwatch data in a more frequent manner, such as every 3 months or every month, to capture gradual changes in the sleep-activity rhythm during early childhood. For infants aged ≤6 months, more frequent observations can also allow us to observe how the predominant rhythm of infants changes from ultradian to circadian by adjusting to the 24-hour cycle in the environment. Another limitation of this study is that although we identified the association of sleep-activity daily rhythm consolidation with early childhood motor development and with large muscle system development in particular, the mechanism behind this association is not clear. Future work should investigate how daily rhythm consolidation and motor development interrelate and contribute to early childhood development.

### Conclusions

In summary, the proposed PML algorithm provides a new method for circadian rhythm analysis and is particularly useful for studying populations whose daily patterns are not regular. In addition, the PML algorithm is applicable to other types of wearable device data in the format of a time series with a nature similar to that of actigraphy; therefore, it can be extended to other types of circadian studies using information such as body temperature, heart rate, and hormone data. Therefore, the PML algorithm can be widely applied to other wearable device studies to help characterize periodic information. Using the proposed method, our study provides novel insights into sleep-activity rhythm development in early childhood. First, in our study, the critical period for the consolidation of sleep-activity rhythms was between 6 and 18 months. This is because at 6 months, 24-hour sleep-wake cycles are formed, but their daily activity patterns are not stabilized, and by the time toddlers reach the age of 18 months, their sleep-activity patterns have consolidated into a fixed pattern with 2 activity peaks and 1 afternoon nap. The period between 6 and 18 months is critical for early childhood sleep-activity rhythm development and consolidation. Second, we identified the association between the consolidation of daily rhythms and early childhood motor development and large muscle system development in particular. This association has not been identified in previous studies. Infants with more consolidated circadian rhythms tend to have better motor development assessments. Although the mechanism is not clear, maintaining a regular and stable sleep-activity pattern and maintaining a healthy circadian system are important for healthy physical development in early childhood.

## Abbreviations

FFT: Fast Fourier Transform
MSE: mean squared error
PDMS-2: Peabody Developmental Motor Scales-Second Edition
PML: Penalized Multiband Learning
